# Metabolic Signature of Leukocyte Telomere Length in Elite Male Soccer Players

**DOI:** 10.3389/fmolb.2021.727144

**Published:** 2021-12-16

**Authors:** Shamma Al-Muraikhy, Maha Sellami, Alexander S Domling, Najeha Rizwana, Abdelali Agouni, Fatima Al-Khelaifi, Francesco Donati, Francesco Botre, Ilhame Diboun, Mohamed A Elrayess

**Affiliations:** ^1^ Biomedical Research Center, Qatar University, Doha, Qatar; ^2^ Department of Drug Design, University of Groningen, Groningen, Netherlands; ^3^ Department of Physical Education (PE), College of Education, Qatar University, Doha, Qatar; ^4^ Department of Pharmaceutical Sciences, College of Pharmacy, QU Health, Qatar University, Doha, Qatar; ^5^ Biomedical and Pharmaceutical Research Unit (BPRU), QU Health, Qatar University, Doha, Qatar; ^6^ Anti-Doping Lab Qatar, Doha, Qatar; ^7^ Laboratorio Antidoping, Federazione Medico Sportiva Italiana, Rome, Italy; ^8^ College of Health and Life Sciences, Hamad Bin Khalifa University (HBKU), Doha, Qatar

**Keywords:** telomere length, elite athletes, soccer, aging, metabolomics

## Abstract

**Introduction:** Biological aging is associated with changes in the metabolic pathways. Leukocyte telomere length (LTL) is a predictive marker of biological aging; however, the underlying metabolic pathways remain largely unknown. The aim of this study was to investigate the metabolic alterations and identify the metabolic predictors of LTL in elite male soccer players.

**Methods:** Levels of 837 blood metabolites and LTL were measured in 126 young elite male soccer players who tested negative for doping abuse at anti-doping laboratory in Italy. Multivariate analysis using orthogonal partial least squares (OPLS), univariate linear models and enrichment analyses were conducted to identify metabolites and metabolic pathways associated with LTL. Generalized linear model followed by receiver operating characteristic (ROC) analysis were conducted to identify top metabolites predictive of LTL.

**Results:** Sixty-seven metabolites and seven metabolic pathways showed significant associations with LTL. Among enriched pathways, lysophospholipids, benzoate metabolites, and glycine/serine/threonine metabolites were elevated with longer LTL. Conversely, monoacylglycerols, sphingolipid metabolites, long chain fatty acids and polyunsaturated fatty acids were enriched with shorter telomeres. ROC analysis revealed eight metabolites that best predict LTL, including glutamine, N-acetylglutamine, xanthine, beta-sitosterol, N2-acetyllysine, stearoyl-arachidonoyl-glycerol (18:0/20:4), N-acetylserine and 3-7-dimethylurate with AUC of 0.75 (0.64–0.87, *p* < 0.0001).

**Conclusion:** This study characterized the metabolic activity in relation to telomere length in elite soccer players. Investigating the functional relevance of these associations could provide a better understanding of exercise physiology and pathophysiology of elite athletes.

## Introduction

Telomeres are repetitive non-coding DNA sequences located at the end of chromosomes, which protect against DNA damage and preserve genome integrity as they shorten during cell division (Blackburn and Gall, 1978; Blackburn et al., 2006). When the mean telomere length reaches a critical value, replicative senescence and cell death occurs. Therefore, telomere length is regarded as a marker of biological aging (Blackburn, 2001; Campisi and d'Adda di Fagagna, 2007; Abdallah et al., 2009). Short leukocyte telomere length (LTL) has been associated with age (Lindsey et al., 1991; Slagboom et al., 1994; Abdallah et al., 2009; Broer et al., 2013) as well as multiple age-related diseases such as diabetes (Jeanclos et al., 1998; Fitzpatrick et al., 2007; Zhao et al., 2013), cardiovascular disease (Brouilette et al., 2003; Brouilette et al., 2007; Fitzpatrick et al., 2007; Maubaret et al., 2010; Haycock et al., 2014; Rehkopf et al., 2016) and dementia (Martin-Ruiz et al., 2006; Honig et al., 2012). Short LTL was also linked to increased risk of mortality (Cawthon et al., 2003; Bakaysa et al., 2007; Kimura et al., 2008; Ehrlenbach et al., 2009; Fitzpatrick et al., 2011; Deelen et al., 2014; Rode et al., 2015; Marioni et al., 2018), although such association was not supported by other studies (Martin-Ruiz et al., 2005; Bischoff et al., 2006; Harris et al., 2006; Njajou et al., 2009; Houben et al., 2011; Strandberg et al., 2011). Despite the large number of studies establishing the link between LTL and wellbeing, the molecular pathways underlying these associations are still largely unknown.

Various alterations of metabolic pathways constitute key features of longevity (Gonzalez-Covarrubias et al., 2013; Montoliu et al., 2014; Cheng et al., 2015). Metabolic changes were also linked with age-related diseases, such as type-2 diabetes, atherosclerosis, cancer, and Alzheimer’s disease (Matsumoto et al., 2007; Suhre et al., 2010; Oresic et al., 2011; Cao et al., 2012; Wang-Sattler et al., 2012; Yu et al., 2012; Floegel et al., 2013; Liu et al., 2017; Al-Khelaifi et al., 2018; Al-Khelaifi et al., 2019; Al-Sulaiti et al., 2019; Ilhame et al., 2020; Mohamed et al., 2020). However, a limited number of studies investigated metabolic pathways associated with LTL (Menni et al., 2013; Zhao et al., 2014; Zierer et al., 2016). One study investigated the metabolic biomarkers of aging in six thousand individuals from TwinsUK registry and identified of 22 metabolites that strongly correlated with age and related traits, but not with LTL (Menni et al., 2013). Another study of 423 American Indians identified 19 metabolites associated with LTL (Zhao et al., 2014). A more recent study tested the association between 280 blood metabolites and LTL in 3,511 females from TwinsUK and replicated results in the KORA cohort. The results confirmed the association of 1-stearoylglycerophosphoinositol and 1-palmitoylglycerophosphoinositol with LTL, suggesting involvement of fatty acid metabolism and particularly membrane composition in biological aging. The study also reported the association of gamma-glutamyltyrosine and gamma-glutamylphenylalanine with LTL, suggesting the involvement of the glutathione cycle and markers of increased oxidative stress (Zierer et al., 2016).

Exercise training was shown to reduce the rate of telomere shortening during the aging process (Shammas, 2011; Balan et al., 2018). Studies have suggested that regular exercise training is associated with longer telomeres (Arsenis et al., 2017); however, the metabolic signature of telomere length in long-term exercising elite endurance athletes (such as soccer players) has not been described. In the current study, an untargeted metabolomics approach was used to investigate the association between LTL and 837 serum metabolites in 126 young male elite soccer players and the metabolic pathways associated with LTL.

## Methods

### Cohort

126 young elite male soccer players who participated in national or international sports events and tested negative for doping substances at anti-doping laboratories in Italy were included in this study. Metabolomics study utilized spare serum samples collected for human growth hormone anti-doping tests. Briefly, samples were delivered to the anti-doing laboratory within 36 h under cooling conditions. Once received, samples were immediately centrifuged to separate the serum and then stored at −20°C until analysis. Due to the strict anonymization process undertaken by anti-doping laboratories and as per study’s ethics, only information related to the type of sport and athlete’s gender were available to researchers. All other information were not available, including age, ethnicity, or the time of recruitment (pre or post exercise). This study was performed in line with the World Medical Association Declaration of Helsinki–Ethical Principles for medical research involving human subjects. All protocols were approved by the Institutional Research Board of Qatar University (QU-IRB 1277-E/20). All participants consented for the use of their samples for research.

### Metabolomics

Metabolic profiling of participants’ serum samples was conducted using established protocols at Metabolon’s established protocols using a Waters ACQUITY ultra-performance liquid chromatography (UPLC) and a Thermo Scientific Q-Exactive high resolution/accurate mass spectrometer interfaced with a heated electrospray ionization (HESI-II) source and Orbitrap mass analyzer operated at 35,000 mass resolution. The detailed description of the liquid chromatography-mass spectrometry (LC-MS) methodology was previously reported (Evans et al., 2014). Briefly, 100 μL of sample was used for each analysis. Small molecules were extracted in an 80% methanol solution containing recovery standards. The resulting extract was divided into five fractions: two for analysis by two separate reverse phase (RP)/UPLC-MS/MS methods with positive ion mode electrospray ionization (ESI), one for analysis by RP/UPLC-MS/MS with negative ion mode ESI, one for analysis by hydrophilic interaction chromatography (HILIC)/UPLC-MS/MS with negative ion mode ESI, and one sample was reserved for backup. On average, 1,009 features per sample (ranges from 906 to 1,038) are measured above the detection limits (Kong and Hernandez-Ferrer, 2020). Raw data were extracted, peak-identified and quality control processed using Metabolon’s hardware and software (DeHaven et al., 2012). Compounds were identified by comparison to library entries of purified standards or recurrent unknown entities with more than 3,300 commercially available purified standard compounds. Library matches for each compound were checked for each sample and corrected if necessary (Evans et al., 2014). Further details of raw data extraction and types of quality control samples and the parameters used to assure data collection quality were previously described (Montrose et al., 2012).

### Measurement of Telomere Length

DNA was extracted from whole blood using DNeasy Blood and Tissue kit according to manufacturer’s instructions (Qiagen, Germany). Nanodrop was used to assess the concentration/quality of DNA. The average LTL in extracted DNA samples were assessed using Absolute Human Telomere Length Quantification qPCR Assay Kit according to manufacturer’s instructions (ScienCell, United States). The kit includes telomere primer set that amplifies telomere sequences, a single copy reference region for data normalization and a reference genomic DNA sample with known telomere length as a reference for calculating the telomere length of target samples.

### Statistical Analysis

Metabolomics data were log-transformed to ensure normality of distribution. Batch correction was performed by Metabolon by rescaling the median of each metabolite to 1. LTL was log transformed to alleviate the original skewing of the distribution. Principal component analysis (PCA) was performed as an exploratory approach for finding out the main factors contributing to the observed variation in the data. Orthogonal partial least square (OPLS) was performed to identify components that best differentiate LTL whilst dissecting orthogonal components that do not differentiate LTL. Both PCA and OPLS were run using SIMCA 16 with the default metabolite-wise metabolite missingness threshold of 50%. Linear models for association analysis were run using the R statistical package (version 2.14, www.r-project.org/) to identify metabolites associated with LTL. The model also corrected for hemolysis levels (determined visually by Metabolon) and PCs from PCA analysis. Function enrichment analysis was performed using the one tailed Wilcoxon sum of the ranks test**.** The LTL variable was then categorized into low (below mean) and high (above mean) levels. A logistic regression (or Generalised linear model based on the Binomial family) was then used together with a step-wise procedure to determine a subset of metabolites (of those with the nominal *p* value ≤ 0.05 from the linear model) that best predicts the categorized LTL (Y variable). Using SPSS statistical package (version 27), the receiver operating characteristic (ROC) analysis was used to assess the discriminatory capacity of the identified subset of metabolites. Kyoto Encyclopedia of Genes and Genomes (KEGG) pathways were utilized in order to gain further insight into the biochemistry of identified metabolites.

## Results

### Multivariate Analysis of Metabolomics Data of Elite Soccer Players

**FIGURE 1:**
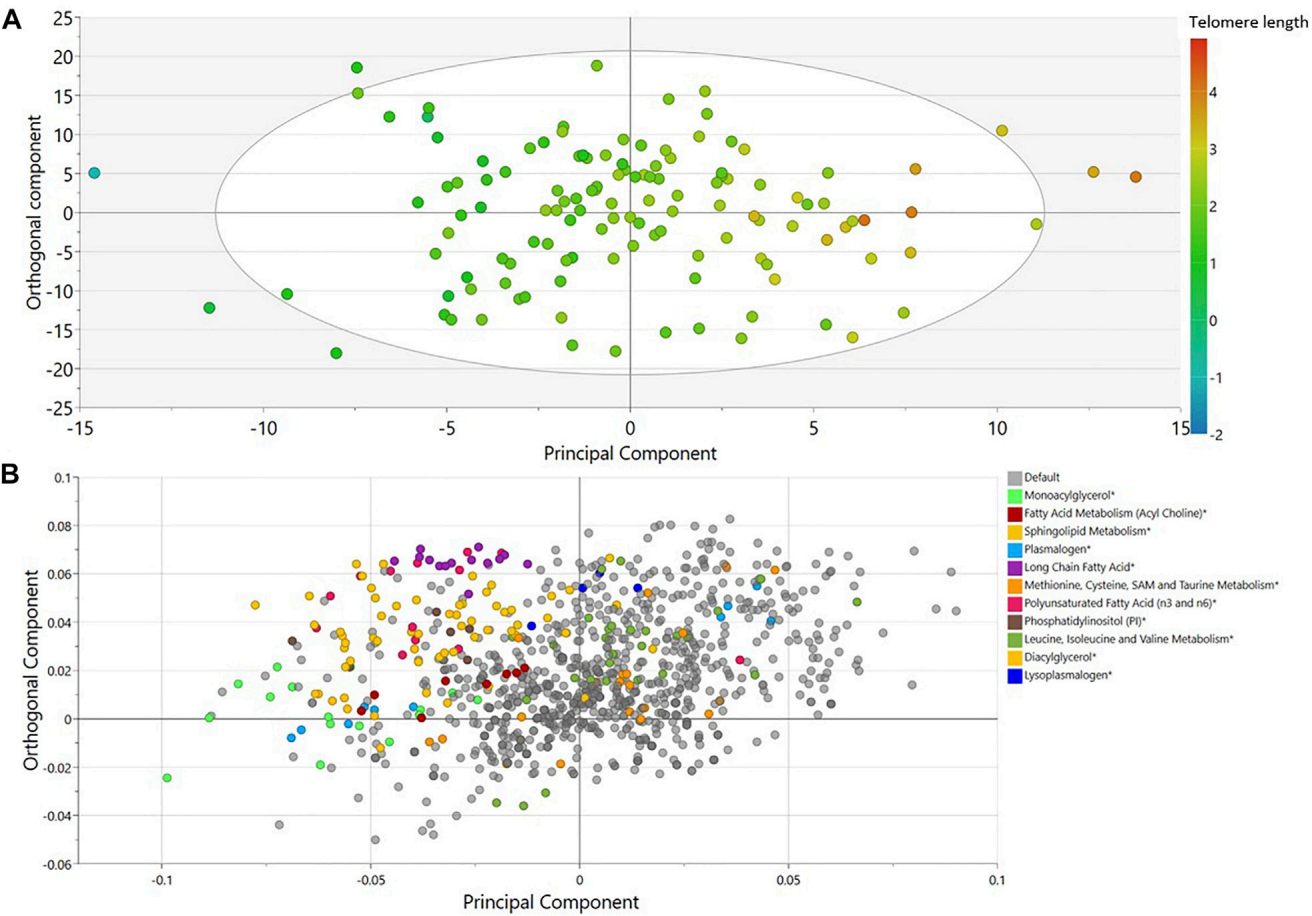
OPLS model of metabolites associated with LTL in elite male soccer players. **(A)** Score plot showing the class-discriminatory component 1 (*x*-axis) versus orthogonal component (*y*-axis) for LTL. **(B)** The corresponding loading plots showing top associated metabolites differentiating short from long LTL. Sub-pathway categories enriched in significant metabolites from regression analysis are shown in color.

Non-targeted metabolomics was applied to determine the metabolic signatures of 126 elite male soccer players. An OPLS analysis revealed one class-discriminatory component accounting for 69% of the variation in the LTL data (Figure 1A). The corresponding loading score, shown in Figure 1B, suggests enrichment of lysophosolipids, benzoate metabolites and glycine/serine/threonine metabolites with longer LTL, whereas monoacylglycerols, long change fatty acids, polyunsaturated fatty acids and sphingolipids were enriched with shorter LTL.

### Univariate Association and Function Enrichment Analyses

**FIGURE 2:**
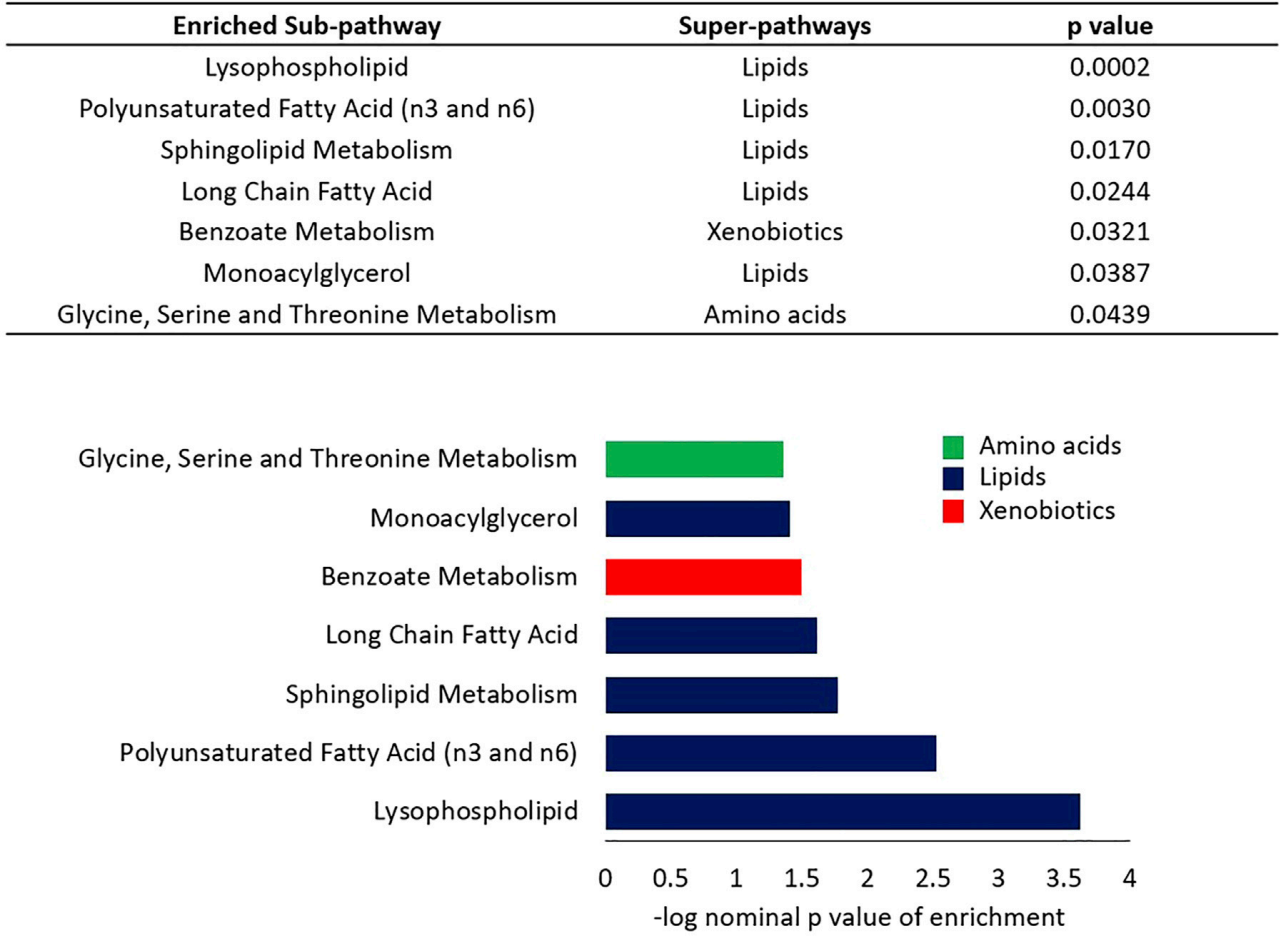
Enrichment analysis of sub-pathways based on regression nominal *p* value. Wilcoxon sum of the ranks test was used to assess the probability of the observed ranks of sub-pathway metabolites when ordered by nominal *p* values from the linear model analysis. Colors indicate the super-pathways of enriched sub-pathways.

**FIGURE 3:**
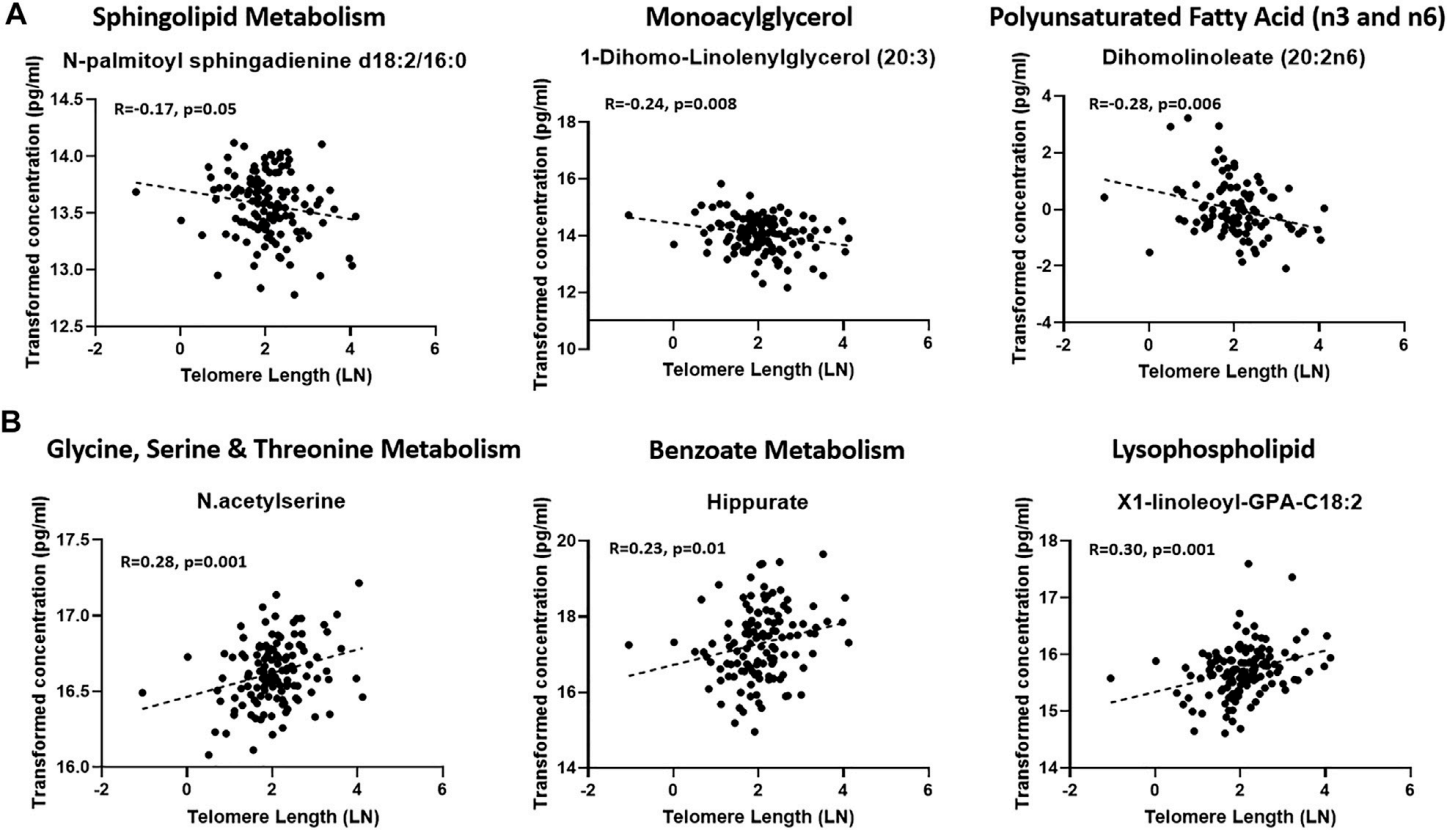
Scatter plots of representatives of enriched pathways that were reduced **(A)** or increased **(B)** with LTL.

A linear model was used to assess the significance of metabolite-associations with the LTL after correcting for hemolysis levels, PC1 and PC2. Sixty-seven metabolites associated with LTL (*p* < 0.05) were identified and their associated pathways listed (Table 1). Enrichment analysis confirmed an over-representation of lysophosolipids, benzoate metabolites and glycine/serine/threonine metabolites with longer LTL, and monoacylglycerols, long change fatty acids, polyunsaturated fatty acids and sphingolipids with shorter LTL (Figure 2). Scatter plots of metabolites representing these pathways, which either decreased (Figure 3A) or increased (Figure 3B) with LTL, are shown in Figure 3.

**TABLE 1:** Metabolites associated with TL after correcting for PCs and hemolysis.

Metabolite	Sub-pathway	Super-pathway	Estimate	SE	Nominal *p* value
Quinolinate	Nicotinate and Nicotinamide Metabolism	Cofactors and Vitamins	−0.22	0.07	0.002
N-Palmitoyl-Sphingadienine (D18:2-16:0)	Sphingolipid Metabolism	Lipid	−0.09	0.03	0.003
Hexadecadienoate (16:2n6)	Polyunsaturated Fatty Acid (n3 and n6)	Lipid	−0.19	0.07	0.005
10-Nonadecenoate (19:1n9)	Long Chain Fatty Acid	Lipid	−0.15	0.05	0.006
4-Hydroxycinnamate-Sulfate	Tyrosine Metabolism	Amino Acid	−0.32	0.09	0.006
1-linoleoyl-GPA (18:2)	Lysophospholipid	Lipid	0.12	0.04	0.006
Stearoyl sphingomyelin (d18:1/18:0)	Sphingolipid Metabolism	Lipid	−0.06	0.02	0.008
3-methoxycatechol sulfate 2	Benzoate Metabolism	Xenobiotics	0.25	0.09	0.009
10-heptadecenoate (17:1n7)	Long Chain Fatty Acid	Lipid	−0.15	0.06	0.01
1-linolenoylglycerol (18:3)	Monoacylglycerol	Lipid	−0.14	0.05	0.011
N-Acetylserine	*Glycine*, Serine and Threonine Metabolism	Amino Acid	0.06	0.02	0.011
Gentisate	Tyrosine Metabolism	Amino Acid	0.39	0.14	0.012
Hippurate	Benzoate Metabolism	Xenobiotics	0.25	0.1	0.012
1-linoleoyl-GPG (18:2)	Lysophospholipid	Lipid	0.11	0.04	0.013
Sedoheptulose	Pentose Metabolism	Carbohydrate	0.15	0.06	0.014
Ceramide (d18:1/14:0, d16:1/16:0)	Ceramides	Lipid	−0.15	0.06	0.015
Glycodeoxycholate	Secondary Bile Acid Metabolism	Lipid	0.24	0.1	0.016
Sebacate (C10-DC)	Fatty Acid, Dicarboxylate	Lipid	0.18	0.07	0.017
Sarcosine	*Glycine*, Serine and Threonine Metabolism	Amino Acid	0.1	0.04	0.017
dihomolinoleate (20:2n6)	Polyunsaturated Fatty Acid (n3 and n6)	Lipid	−0.11	0.05	0.017
Xanthine	Purine Metabolism, (Hypo)Xanthine/Inosine containing	Nucleotide	0.08	0.03	0.017
Dimethylglycine	*Glycine*, Serine and Threonine Metabolism	Amino Acid	0.07	0.03	0.017
1-arachidonoyl-GPC (20:4)	Lysophospholipid	Lipid	−0.06	0.02	0.017
1-palmitoylglycerol (16:0)	Lysophospholipid	Lipid	0.12	0.05	0.017
Glycocholate	Primary Bile Acid Metabolism	Lipid	0.25	0.1	0.018
1-stearoyl-GPC (18:0)	Lysophospholipid	Lipid	−0.04	0.02	0.018
1-linoleoyl-GPi (18:1)	Lysophospholipid	Lipid	0.16	0.07	0.019
1-stearoyl-2-arachidonoyl-GPI (18:0/20:4)	Phosphatidylcholine (PC)	Lipid	−0.04	0.02	0.02
1-stearoyl-GPS (18:0)	Lysophospholipid	Lipid	0.17	0.07	0.02
Gamma-Glutamylglutamine	Gamma-glutamyl Amino Acid	Peptide	−0.14	0.06	0.021
15-Methylpalmitate	Fatty Acid, Branched	Lipid	−0.11	0.05	0.022
Beta-Hydroxyisovalerate	Leucine, Isoleucine and Valine Metabolism	Amino Acid	0.12	0.05	0.023
Docosapentaenoate (DPA; 22:5n3)	Polyunsaturated Fatty Acid (n3 and n6)	Lipid	−0.12	0.05	0.024
N-Acetyl-3-Methylhistidine	Histidine Metabolism	Amino Acid	0.31	0.13	0.024
Sphingomyelin (d18:1/20:0, d16:1/22:0)	Sphingolipid Metabolism	Lipid	−0.04	0.02	0.025
Palmitoleate (16:1n7)	Long Chain Fatty Acid	Lipid	−0.17	0.07	0.026
Sphingomyelin (d18:1/22:1, d18:2/22:0, d16:1/24:1)	Sphingolipid Metabolism	Lipid	−0.03	0.01	0.026
1-stearoyl-2-arachidonoyl-GPI (18:0/20:4)	Phosphatidylinositol (PI)	Lipid	−0.05	0.02	0.029
margarate (17:0)	Long Chain Fatty Acid	Lipid	−0.1	0.04	0.029
X5alpha-Androstan-3alpha-17beta-Diol-Disulfate	Androgenic Steroids	Lipid	0.25	0.11	0.03
1-Oleoyl-Gpg (18:1)	Lysophospholipid	Lipid	0.13	0.06	0.031
1-Palmitoyl-Gpg (16:0)	Lysophospholipid	Lipid	0.09	0.04	0.032
4-Acetaminophen-Sulfate	Drug	Xenobiotics	1.77	0.55	0.032
1-Dihomo-Linolenylglycerol (20:3)	Monoacylglycerol	Lipid	−0.14	0.07	0.033
1-Arachidonylglycerol (20:4)	Monoacylglycerol	Lipid	−0.12	0.05	0.034
3-phenylpropionate (hydrocinnamate)	Benzoate Metabolism	Xenobiotics	0.19	0.09	0.036
Glycerate	Glycolysis, Gluconeogenesis, and Pyruvate Metabolism	Carbohydrate	0.05	0.02	0.036
Glutamine	Glutamate Metabolism	Amino Acid	−0.06	0.03	0.036
Lactosyl-N-Behenoyl-Sphingosine (D18:1/22:0)	Sphingolipid Metabolism	Lipid	0.1	0.05	0.036
1-Palmitoyl-Gpi (16:0)	Lysophospholipid	Lipid	0.13	0.06	0.038
1-2-Dilinoleoyl-Gpc (18:2-18:2)	Phosphatidylcholine (PC)	Lipid	0.06	0.03	0.039
1-Palmitoleoylglycerol (16:1)	Monoacylglycerol	Lipid	−0.1	0.05	0.04
Glycochenodeoxycholate glucuronide (1)	Primary Bile Acid Metabolism	Lipid	0.2	0.1	0.041
3-(3-hydroxyphenyl)propionate	Benzoate Metabolism	Xenobiotics	0.23	0.11	0.041
N-Acetylglutamate	Glutamate Metabolism	Amino Acid	0.11	0.05	0.041
sphingomyelin (d18:1/22:2, d18:2/22:1, d16:1/24:2)	Sphingolipid Metabolism	Lipid	−0.05	0.02	0.042
X3-Hydroxyhippurate	Benzoate Metabolism	Xenobiotics	0.26	0.12	0.042
Sphingomyelin (D18:2-23:1)	Sphingolipid Metabolism	Lipid	−0.05	0.02	0.043
Arachidonate (20:4n6)	Polyunsaturated Fatty Acid (n3 and n6)	Lipid	−0.05	0.03	0.043
4-Acetylphenol-Sulfate	Drug	Xenobiotics	0.24	0.11	0.043
Cotinine-N-Oxide	Tobacco Metabolite	Xenobiotics	−0.25	0.11	0.046
Sphingomyelin (d18:1/21:0, d17:1/22:0, d16:1/23:0)	Sphingolipid Metabolism	Lipid	−0.05	0.03	0.046
Hypoxanthine	Purine Metabolism, (Hypo)Xanthine/Inosine containing	Nucleotide	0.08	0.04	0.048
1-(1-enyl-palmitoyl)-2-arachidonoyl-GPE (P-16:0/20:4)	Plasmalogen	Lipid	−0.08	0.04	0.048
dihomolinolenate (20:3n3 or 3n6)	Polyunsaturated Fatty Acid (n3 and n6)	Lipid	−0.07	0.04	0.048
Stearate (18:0)	Long Chain Fatty Acid	Lipid	−0.05	0.03	0.05
Betaine	*Glycine*, Serine and Threonine Metabolism	Amino Acid	0.04	0.02	0.05

### Predictive Metabolites of LTL

**FIGURE 4:**
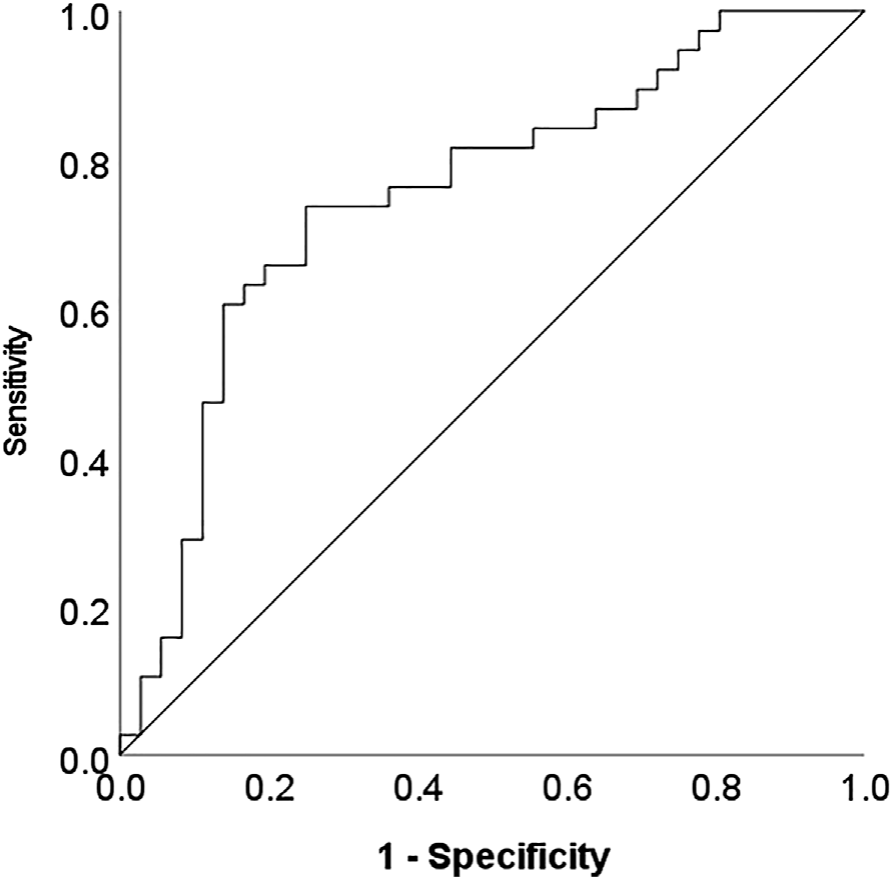
ROC curve analysis indicating the discriminatory power of a subset of metabolites differentiating short and long LTL (below/above mean respectively).

A Generalized Linear Model featuring the mean-dichotomized LTL as the y-variable and top metabolites from the linear model as explanatory variables revealed a set of eight best predictive metabolites, including glutamine, N-acetylglutamine, xanthine, beta-sitosterol, N2-acetyllysine, stearoyl-arachidonoyl-glycerol (18:0/20:4), N-acetylserine and 3-7-dimethylurate. The area under curve (AUC) value from the ROC curve analysis was 0.75 (0.64–0.87, *p* < 0.0001) (Figure 4).

## Discussion

Studies performed in human cell lines and various model organisms, including non-human primates, have revealed several longevity regulatory pathways involved in genome stability, energy metabolism and self-recognition (Riera et al., 2016; Campisi et al., 2019). Together, these pathways promote somatic maintenance via normal stem cell function and activation of autophagy, defense mechanisms against infectious agents, and survival pathways, while attenuating pro-inflammatory mediators, deregulated cell growth and senescence (Riera et al., 2016; Campisi et al., 2019). Previous studies have shown that exercise-mediated telomere preservation is associated with activation of specific metabolic pathways that could play a major role in disease prevention through exercise (Denham et al., 2016). However, several questions remain unanswered about how these longevity regulatory pathways are interconnected, or even hierarchical, to determine the telomere attrition through time and how they are related to regular practice of sport. In this study, the metabolic predictors of LTL were determined in 126 young elite male soccer players. Several metabolites and metabolic pathways were increased or decreased with LTL. Among these, top predictors of LTL were determined, providing a better understanding of exercise physiology and pathophysiology of elite athletes.

### Changes in Various Lipids Are Associated With LTL

Whereas levels of lysophospholipids were elevated with longer LTL, other lipid species, including monoacylglycerols, long chain fatty acids, polyunsaturated fatty acid (n3 and n6) and sphingolipids, were reduced. Previous studies have shown that blood lysophosphatidylcholine levels tend to decrease with age (Johnson and Stolzing, 2019). Our data are in agreement with these findings as lower levels of lysophatidylcholines were associated with shorter LTL in elite soccer players. The blood profile of other phospholipids and sphingolipids was also shown to change with age, suggesting that blood lipids provide a rich source of biomarkers of human aging and potently regulators of aging and lifespan. Plasma lipidomics of 11 mammalian species ranging in longevity from 3.5 to 120 years has identified a predictive metabolic signature of the mammal’s lifespan, including long‐chain free fatty acids, lipid peroxidation index, and lipid peroxidation‐derived content, which were inversely correlated with longevity (Jové et al., 2013). These data confirm our findings as elevated levels of long chain fatty acids, polyunsaturated fatty acid (n3 and n6) and sphingolipids were associated with shorter telomeres. Further evidence linking lipid metabolism to aging came from animals with extreme longevity. The very long‐lived (over 500 years) quahog clam *Arctica islandica* was shown to have an exceptional resistance to lipid peroxidation in mitochondrial membranes (Munro and Blier, 2012). The lens membranes of the bowhead whale that can live longer than 200 years are highly enriched with phospholipids, providing resistance to the age‐related cataracts disease (Borchman et al., 2017). Hence, our emerging data confirm the important role of lipids in longevity and suggest the ability of lipid‐based interventions to modulate longevity in model organisms (Huang et al., 2014).

### Changes in Benzoate and Glycine Metabolism Are Associated With Longer LTL

Our data showed elevation in betaine (dimethylglycine precursor), dimethylglycine (sarcosine precursor), sarcosine (glycine precursor), hippurate (glycine product) and 3-hydroxyhippurate (hippurate product), suggesting increased hippurate synthesis from glycine in athletes with longer LTL (Pallister et al., 2017; Tahir et al., 2019). Hippurate is a glycine conjugate of benzoate and is related to the gut microbiome and correlated to kidney function. Previous studies have identified higher hippurate levels in younger subjects (Lees et al., 2013; Adav and Wang, 2021), suggesting that elevated hippurate synthesis could be a biomarker of slower aging. The functional relevance of these metabolic changes requires further investigation.

### Metabolic Predictors of LTL

Our data has also identified a metabolic classifier based on eight metabolites that can predict LTL in our cohort. Selected metabolites included four amino acids (glutamine, N-acetylglutamine, N-acetylserine, N2.acetyllysine), two lipids (stearoyl-arachidonoyl-glycerol (18:0/20:4), beta-sitosterol) and one nucleotide (xanthine). In this model, reduced glutamine levels were associated with longer LTL. Previous studies have identified that older subjects have higher glutamine compared to younger subjects (Kaiser et al., 2005), confirming our observation. The classifier also identified that elevated xanthine was associated with increased LTL, confirming a multi-omics study that showed xanthine as a pro-survival metabolite with aberrant mitochondrial function (Gioran et al., 2019). Similarly, our model also suggested that elevated beta-sitosterol is increased with longer LTL. Previous reports have shown that beta-sitosterol provides immunomodulatory, anti-microbial, anti-cancer, anti-inflammatory, lipid-lowering, hepato-protective, protective effect against respiratory diseases, wound-healing, anti-oxidant and anti-diabetic activities (Babu and Jayaraman, 2020). The association of the other metabolites included in the metabolic classifier of LTL was not reported before, therefore requires further confirmation in other cohorts.

### Study Limitations

In this pilot study, the relatively small number of participants and the variation in sample collection, transportation and storage could have affected the results. Additionally, the limited available information of athletes’ anthropometric, physiological, and nutritional data during sampling as well as the resting time since their last exercise has hindered attempts to consider other important potential confounders such as body mass index and diet in our analysis. Furthermore, the use of young elite athletes without inclusion of non-athlete controls has limited the usefulness of the data, although the current study used a “within-subjects” design to test the effect of long-term soccer practice on LTL and metabolic profile without a control group.

Despite all these limitations, our novel data has revealed significant, although differences were only nominally significant, and pronounced alterations in metabolites with LTL in elite male soccer players. However, replication of data in other cohorts with controlled experimental design, including having non-elite athlete controls, is warranted to validate the emerging metabolites set as potential predictive biomarkers of exercise-associated reduced telomere attrition.

## Conclusion

Our novel data suggest that elite soccer players exhibit a unique metabolic signature associated with LTL, including specific metabolites that can best predict LTL. The functional relevance of the emerging data is to highlight the importance of lipids, glutamine, xanthine, beta-sitosterol, among other metabolites, on longevity in response to chronic exercise, potentially through triggering a pro-survival, immunomodulatory and anti-oxidant effect. Confirming the predictive power of these metabolites and their functional relevance in different cohorts could help in understanding the aging process and the metabolic pathways underlying exercise-associated reduced telomere attrition.

## Data Availability

The datasets presented in this study can be found in online repositories. The names of the repository/repositories and accession number(s) can be found below: https://www.ebi.ac.uk/metabolights/, MTBLS2976.
